# Finding specific cut-off values of JADAS-10 and JADAS3-10 for disease activity levels in juvenile idiopathic arthritis: a finnish multicenter study

**DOI:** 10.1186/1546-0096-12-S1-P149

**Published:** 2014-09-17

**Authors:** Maria Backström, Pirjo Tynjälä, Heikki Ylijoki, Kristiina Aalto, Johanna Kärki, Heini Pohjankoski, Paula Keskitalo, Sirja Sard, Maiju Hietanen, Helena Lehto, Tommi Kauko, Paula Vähäsalo

**Affiliations:** 1 Department of Pediatrics, Vaasa Central Hospital, Vaasa, Finland; 2 Department of Pediatrics South-Karelian Central Hospital, Lappeenranta, Finland; 3 Poison Information Center, Helsinki University Central Hospital, Helsinki, Finland; 4 Department of Pediatrics, Satakunta Central Hospital, Pori, Finland; 5 Children`s Hospital, Rheumatology Unit, Helsinki University Central Hospital, Helsinki, Finland; 6 Department of Pediatrics, Kanta-Häme Central Hospital, Hämeenlinna, Finland; 7 Department of Pediatrics, Päijät-Häme Central Hospital, Hämeenlinna, Finland; 8 Medical Research Center Oulu, Department of Pediatrics, Oulu University Hospital and University of Oulu, Oulu, Finland; 9 Department of Biostatistics, University of Turku, Turku, Finland

## Introduction

It’s crucial to observe the changes in disease activity in JIA in order to investigate reliably the effects of the new treatments and to optimize their use. Several tools for outcome measurements have been developed. Juvenile Arthritis Disease Activity Score (JADAS) is an independent measure of the treatment response and enables the comparison between cohorts. However, the use of ESR restricts the feasibility of JADAS. That’s why a JADAS index excluding ESR has been tested (JADAS3). In order to simplify the interpretation of the JADAS score, efforts have been made to define cut-off values of JADAS for low and high diease activity. Cut-off values of JADAS for disease activity levels, as defined by ACR, have not been developed. Neither have any cut-off values for JADAS3 been defined.

## Objectives

Our aim was to define the cut-off values for low, moderate, and high disease activity of JADAS-10 and JADAS3-10.

## Methods

In a multicenter study consisting 20% of all patients with juvenile idiopathic arthritis in Finland (n=514), data on last registered visits and on visits fulfilling the criteria for high disease activity were obtained. JADAS-10 and JADAS3-10 were calculated, and the cut-off values of both of these scores were determined by 3 different ROC-based statistical methods.

## Results

Of 514 patients included, 65.5% were females and 54.1% had polyarticular disease. The most suitable method for determining cut-off values was the 90% specificity index. The cut-off value for low disease activity was 0.2 for both JADAS-10 and JADAS3-10 in both oligoarticular and polyarticular disease. In oligoarticular disease, the cut-off value for moderate disease activity was 2.8 for JADAS-10 and 2.4 for JADAS3-10 and in polyarticular disease 4.1 for both JADAS-10 and JADAS3-10. In patients with polyarticular disease, the cut-off value for high disease activity of JADAS-10 and JADAS3-10 was 16.3 and 16.0.

## Conclusion

Cut-off values for low, moderate and high disease activity were defined. In clinical setting, research and quality control, JADAS3-10 can be used instead of JADAS-10. In the future, uniform, clinical disease activity levels need to be set. Valid and robust cut-off values of disease activity levels can guide both a clinician and a researcher, and equip in benchmarking.

## Disclosure of interest

M. Backström grant / research support from: Pfizer, Abbvie, Roche, and, consultant for: Pfizer, Abbvie. P. Tynjälä: none declared. H. Ylijoki: None declared. K. Aalto grant / research support from: Abvie (Abbot), BMS, Roche, Sobi, Wyeth, and consultant for: Roche, Sobi. J. Kärki: None declared. H. Pohjankoski: None declared. P. Keskitalo: None declared. S. Sard: None declared. M. Hietanen: None declared. H. Lehto: None declared. T. Kauko: None declared. P. Vähäsalo grant / research support from: Pfizer, Abbvie, MSD, UCB, and, consultant for: Abbvie, Pfizer, Roche.

